# *Serendipita indica*-dominated synthetic microbial consortia enhanced tartary buckwheat growth and improved its tolerance to drought stress

**DOI:** 10.3389/fmicb.2025.1562341

**Published:** 2025-03-19

**Authors:** Shanpu Zhong, Wenjing Wang, Wuyao Tang, Xingmei Zhou, Tongliang Bu, Zizhong Tang, Qingfeng Li

**Affiliations:** College of Life Sciences, Sichuan Agricultural University, Ya’an, China

**Keywords:** *Fagopyrum tataricum*, microbial consortium, plant growth promotion, *Serendipita indica*, soil improvement

## Abstract

The cultivation of tartary buckwheat serves dual roles, offering health benefits and nutritional advantages. Nonetheless, its cultivation is challenged by issues such as soil degradation and climatic drought. Plant growth-promoting (PGP) microorganisms hold promise for addressing these challenges. In this study, we investigated the effects of *Serendipita indica* inoculation on the root-associated microbial communities of tartary buckwheat. Additionally, we used *S. indica* to construct synthetic microbial consortia, and their role in promoting the growth and enhancing the drought resistance of tartary buckwheat was evaluated. This study found that the colonization of *S. indica* in tartary buckwheat promoted the enrichment of beneficial microorganisms such as Actinobacteriota, *Sphingomonas*, and *Mortierella*, while reducing the relative abundance of pathogenic genera including *Cladosporium*, *Alternaria*, and *Acremonium*. In addition, the inoculation of the microbial consortia significantly promoted the photosynthesis and biomass accumulation of tartary buckwheat, while also improving soil structure and fertility. Under drought conditions, introducing microbial groups markedly boosted root development, lowered the density of stomata and rate of transpiration in tartary buckwheat leaves, and decreased H_2_O_2_ and Malondialdehyde (MDA) levels, thus greatly enhancing tartary buckwheat’s resistance to drought. In conclusion, our findings demonstrated that the microbial consortia constructed with *S. indica* can significantly promote the growth of tartary buckwheat and enhance its drought resistance. However, the specific molecular mechanisms underlying these effects require further investigation in future studies. These findings will provide important theoretical support for the development of novel microbial fertilizers.

## 1 Introduction

Known as the “King of Grains,” tartary buckwheat (*Fagopyrum tataricum*) is a yearly herb from the Polygonaceae family and *Fagopyrum* genus. This substance is abundant in nutrients, rich in proteins, amino acids, and minerals, and also includes medicinal elements like rutin and quercetin. The bioactive elements in tartary buckwheat bestow significant health benefits such as reducing blood sugar, lipid levels, and aging, establishing it as a valuable crop for both health and nutrition (Skrabanja et al., 2001; Huda et al., 2021; Jha et al., 2024; Zamaratskaia et al., 2024). The primary cultivation of tartary buckwheat takes place in the elevated mountain areas of the provinces of Sichuan, Guizhou, and Yunnan in China (Zhu, 2016; Song et al., 2020). However, the cultivation of this plant is hindered by various obstacles, including low soil fertility, dry weather, insufficient irrigation, and soil erosion, all of which significantly impede enhancing its yield and quality. Within conventional farming methods, chemical fertilizers are frequently used to increase tartary buckwheat production. However, excessive use of chemical fertilizers not only exacerbates soil acidification and salinization but also leads to low fertilizer utilization efficiency and soil degradation (Pahalvi et al., 2021; Das et al., 2023). Consequently, it’s crucial to investigate a proficient, eco-friendly, and sustainable method to lessen dependence on chemical fertilizers, with the use of microbial fertilizers anticipated to address these issues.

Microbial fertilizers, also referred to as biofertilizers, are defined as live microbial formulations composed of plant growth-promoting microorganisms or their metabolic byproducts. Microbial fertilizers, in contrast to chemical fertilizers, demonstrate eco-friendly properties. Not only do they significantly boost crop development and yield, but they also better the soil’s composition and aid in nutrient recycling within the soil (Sathya et al., 2016). Vegetation emits secretions from roots into the adjacent soil, luring certain microbes to inhabit the rhizosphere or root surface, thus supplying these microbes with nourishment and living spaces, leading to the formation of a distinct microbial community (Bai et al., 2022). Consequently, microorganisms linked to roots enhance plant development via diverse pathways. As an example, they directly boost plant development through the synthesis of phytohormones, dissolution of phosphorus, nitrogen fixation, or creation of siderophores. On the other hand, they contribute to plant development indirectly by fostering plant resilience and aiding in the defense against pathogens (Benaissa, 2019; Batabyal, 2021). As an illustration, introducing the endophytic bacterium ST-B2 markedly enhances the development of hypocotyls and roots in tartary buckwheat, concurrently boosting the biomass of seedlings (Briatia et al., 2017). Likewise, the application of a biocontrol agent to tartary buckwheat seeds is known to improve leaf photosynthesis and notably increase grain production (Witkowicz et al., 2021). Additionally, a variety of microorganisms that encourage plant growth have the ability to alter the inherent microbial community composition in the root system, foster the proliferation of advantageous microbes, and increase the availability of nutrients in the soil, thus aiding in plant development. As an instance, it was found that using arbuscular mycorrhizal fungi (AMF) controlled the rhizosphere’s bacterial population in *Lotus japonicus*, enhancing bacteria that dissolve inorganic phosphorus and potassium, thereby boosting soil nutrient accessibility (Xu et al., 2023a). Although the majority of research indicates that single-microorganism inoculation enhances plant development, the intricate interplay between root-related microbes frequently restricts the uniformity and steadiness of these impacts (Qiu et al., 2019; Santoyo et al., 2021). As a result, it has been demonstrated that microbial groups possessing various functional characteristics yield more efficient and dependable results in enhancing plant development and soil vitality.

The earlier research demonstrated that *S. indica* stimulates the activation of genes linked to the auxin synthesis route in tartary buckwheat, consequently increasing its biomass. Furthermore, it has been demonstrated that *S. indica* enhances antioxidant enzyme activity in tartary buckwheat leaves, aiding in better drought resistance (Zheng et al., 2023). However, the impact of *S. indica* on the microbial community structure in the rhizosphere of tartary buckwheat remains unclear. Furthermore, studies on the growth-promoting effects of microbial consortia on tartary buckwheat are still limited. In this research, we employed advanced sequencing techniques to explore the variety of microbes in the roots and rhizosphere of tartary buckwheat following its inoculation with *S. indica*. Screening of strains likely to enhance growth was conducted, leading to the creation of synthetic microbial groups containing *S. indica*. Systematic assessments were conducted on how the consortia improved tartary buckwheat’s growth and drought resilience, and its contribution to soil structure and fertility enhancement. The results will offer crucial theoretical backing for creating new microbial fertilizers and bear substantial consequences for the progression of sustainable farming.

## 2 Materials and methods

### 2.1 Fungal and bacterial strains preparation

The *Serendipita indica* strain was donated by Shaoshuai Liu (Beijing Academy of Agricultural and Forestry Sciences, Beijing, China), other strains used in this study were previously isolated by Microbial-Plant Interaction Laboratory (College of Life Sciences, Sichuan Agricultural University) from the rhizosphere and roots of wild buckwheat, and all the strains are shown in Supplementary Figure S1. These included four fungal strains: *Serendipita indica*, *Mortierella alpina* (accession number: ON038743.1), *Ceriporia lacerata* (Accession number: JX623924.1), and *Fusarium equiseti* (Accession number: ON573395.1), as well as three bacterial strains: *Bacillus cereus* (Accession number: KU551246.1), *Rhodobacter sphaeroides* (Accession number: AF547169.1), and *Rhodococcus sphaeroides* (Accession number: ON819749.1). Fungal strains were cultured on Potato Dextrose Agar (PDA) medium at 28°C for 7 days, while bacterial strains were grown on Luria-Bertani (LB) agar plate at 30°C for 3 days. All cultures were stored at 4°C for preservation.

### 2.2 Determination of PGP traits of strains *in vitro*

Based on the method of Khalil et al. (2021), with modifications, strains were inoculated into PDB and LB liquid media containing 150 mg/L tryptophan and incubated at 28°C on a shaker for 7 days. The cultures were then centrifuged at 8,000 rpm for 10 min, and the supernatants were collected to quantify the IAA concentration. NBRIP medium and Aleksandrov agar medium were used to screen for strains that could dissolve phosphate (P) and potassium (K), respectively. The selected strains were then inoculated into the corresponding liquid media and incubated at 28°C on a shaker for 7 days. After incubation, the cultures were centrifuged at 8,000 rpm for 10 min. The concentrations of available phosphorus and soluble potassium in the supernatants were determined using the molybdovanadate method (Lin et al., 2022) and the sodium tetraphenylborate method (Tubino and Torres, 1992) respectively. Following the method of Penrose and Glick (2003), strains were inoculated into DF medium and ADF medium, the latter supplemented with 1-aminocyclopropane-1-carboxylic acid (ACC) as the sole nitrogen source. The cultures were incubated at 28°C for 7 days and sub-cultured for three consecutive generations, the ACC deaminase activity of each strain was subsequently assessed. The nitrogen-fixing ability of each strain was evaluated using nitrogen-free medium (Fu et al., 2022). Siderophore production was detected using Chrome Azurol S (CAS) medium according to the method described by Louden (Louden et al., 2011). Strains were spot-inoculated onto CAS solid medium and incubated at 28°C for 7 days. The presence of an orange-yellow halo surrounding the colonies was observed, indicating the production of the siderophore, and the ratio of the halo diameter (D) to the colony diameter (d) was recorded.

### 2.3 Strains compatibility assay

The biological compatibility among strains was evaluated through antagonistic assays (Anith et al., 2021; Zhou et al., 2022). For bacterial strains, the cross-streak method was employed on LB agar plates, where two different bacterial strains were streaked in a cross pattern and incubated at 28°C for 5 days. The growth of the strains was then observed. The compatibility between fungal and bacterial strains was assessed using the filter paper disk method. A 5-mm fungal mycelial plug was inoculated at the center of a PDA plate, and a 3-mm filter paper disk was placed 2.5 cm away from the center. The disk was inoculated with 10 μL of bacterial suspension, and after absorption, the plates were incubated at 28°C for 5 days to observe the growth interactions. For fungal strains, the dual-culture confrontation method was used. Two 5-mm mycelial plugs from different fungal strains were symmetrically inoculated 2.5 cm from the center of a PDA plate and incubated at 28°C for 5 days. The presence of a distinct inhibition zone between colonies indicated antagonistic interactions between the strains.

### 2.4 Preparation of composite microbial agent and pot experiment

Fungal strains were inoculated into 200 mL of PDB medium and incubated at 28°C with shaking at 120 r/min for 14 days. A spore suspension with a concentration of 2% (*w/v*) (±1 × 10^7^ spores/mL) was prepared using sterile water. Bacterial strains were inoculated into 200 mL of LB medium and incubated at 28°C with shaking at 120 r/min for 7 days. A bacterial suspension with a concentration of 1 × 10^8^ cells/mL was prepared using sterile water. Based on the strain combination results, the microbial suspensions were mixed at a 1:1 volumetric ratio to prepare the composite microbial inoculum for further use. Shriveled tartary buckwheat seeds were removed using the seed liquid selection method, and plump seeds were selected and soaked in warm water at 42°C for 2 h. The microbial inoculum was mixed with soil at a ratio of 1,000 mL/kg, while the control group received distilled water. Each treatment consisted of six pots, with four tartary buckwheat plants per pot.

### 2.5 Measurement of growth phenotypes and photosynthetic parameters in tartary buckwheat

After 1 month of co-cultivation, plant growth parameters of tartary buckwheat were measured, including plant height, leaf length, leaf width, petiole length, fresh and dry weight of stalks, and fresh and dry weight of roots. Plant height, leaf length, leaf width, and petiole length of tartary buckwheat were measured using a ruler. The fresh weights of stalks and roots were determined using an MTQ300 electronic balance (Meilun, Shenzhen, China). The stalks and roots were then dried to a constant weight in an oven at 80°C to measure their dry weight. Each treatment was replicated 10 times. The group exhibiting the best plant growth performance among different strain number combinations was selected for subsequent experiments.

Fresh tartary buckwheat leaves were collected, washed thoroughly with distilled water, and absorbed surface moisture. A 0.1 g sample was weighed and ground into a fine powder under dark conditions. The powdered sample was extracted with 10 mL of acetone-ethanol solution (acetone: ethanol = 2:1, *v/v*) and incubated in darkness for 3 h until the leaf residues were completely bleached. The mixture was then centrifuged, and the supernatant was collected. Using the extraction solution as a blank control, the absorbance at 663 nm and 645 nm was measured with a V-1100D spectrophotometer (MAPADA, Jinan, China). The contents of chlorophyll a, chlorophyll b, and total chlorophyll were calculated using the equations described by Arnon (1949). Leaves from the same node of each plant were selected, and the transpiration rate (E), stomatal conductance (Gs), net photosynthetic rate (A), and intercellular CO₂ concentration (Ci) were measured using the GFS-3000 fluorescence imaging system (WALZ, Nuremberg, Germany).

### 2.6 Measurement of soil physicochemical properties and enzyme activities

After harvesting the plants, soil samples from the 5–10 cm depth layer were collected using a soil ring knife. Roots of tartary buckwheat, stones, and other debris were removed, and the soil was weighed and then dried in an oven at 105°C until a constant weight was achieved, and then soil bulk density, porosity, and water content were calculated (Wang J. et al., 2022). The distribution of soil aggregates was determined using the dry sieving method (Nahidan and Nourbakhsh, 2018). After air-drying, 100 g of soil was accurately weighed and placed on the top layer of a nested sieve set with mesh sizes of 2 mm, 1 mm, 0.5 mm, and 0.25 mm, arranged from top to bottom. The soil was sieved for 5 min, and the soil retained on each sieve was collected and weighed to calculate the percentage of soil aggregates in each size fraction. Air-dried soil was passed through a 2 mm sieve for further analysis. Soil organic matter was determined using the potassium dichromate method (Xie et al., 2021), total nitrogen was measured using the Kjeldahl method (Guo et al., 2024), and available phosphorus (AP) and available potassium (AK) were analyzed using the sodium bicarbonate extraction-molybdenum antimony blue colorimetric method (Olsen, 1954) and flame photometry (Chen et al., 2024), respectively. The activities of soil urease (S-UE), sucrase (S-SC), and alkaline phosphatase (S-AKP/ALP) were measured using assay kits provided by Beijing Boxbio Science & Technology Co., Ltd.

### 2.7 Rhizosphere soil and root samples DNA extraction, amplification, and pyrosequencing

DNA from the samples was extracted using the TGuide S96 DNA extraction kit (DP812, Tiangen Biotech Co., Ltd., China). The integrity and quantity of the extracted DNA were assessed using 1.8% agarose gel electrophoresis and the LabChip GX Touch system (cls137031/E, PerkinElmer, Inc., America). For the bacterial sequencing library, we targeted the16S rRNA V3-V4 gene region (338F: 5′-ACTCCTACGGAGGCAGCA-3′, 806R:5′-GGACTACHVGGGTWTCTAAT-3′). For the fungal sequencing library, we targeted the ITS1F (5′-CTTGGTCATTTAGAGGAAGTAA-3′) and ITS1E (5′-GCTGCGTTCTTCATCGATGC-3′). The PCR products were examined by electrophoresis on a 1.8% agarose gel and purified using the OMEGA DNA purification kit. Pyrosequencing was performed on the Illumina NovaSeq platform (NovaSeq 6000, Illumina, United States).

### 2.8 Analysis of microbial community structure and diversity

The obtained sequences were trimmed and optimized and then clustered into operational taxonomic units (OTUs) at a 97% similarity level. OTUs were filtered using a threshold of 0.005% of the total sequence count. Species annotation was carried out using the Silva database (Release 132^1^) for the bacterial sequences and the Unite database (Release 8.0^2^) for the fungal sequences based on the Blast algorithm, calculated by QIIME2 software (Version 2020.6). Alpha and beta diversity were evaluated by QIIME2 software (version 2020.6) based on OTU normalized data. Inter-group differential species analysis was performed using the Python LEfSe package, and linear discriminant analysis (LDA) was employed to estimate the effect size of species abundance on group differences.

### 2.9 Drought treatment and phenotyping of tartary buckwheat

After 30 days of co-cultivation, tartary buckwheat plants in each treatment group were subjected to natural drought stress for 3 days until their leaves witted, stomatal density in the leaves and root phenotypic parameters were then measured. Leaves from the same node of each plant were selected, apply transparent nail polish to the same position on the abaxial epidermis of tartary buckwheat leaves. After the nail polish dried, the resulting film was carefully peeled off with tweezers and transferred onto a glass slide. Stomatal numbers and opening/closing statuses were then observed under a microscope. The root phenotypic traits of tartary buckwheat were analyzed using a Microtek root scanning system (MRS-9600TFU2L, Shanghai Microtek Technology Co., Ltd., China).

### 2.10 Determination of antioxidant enzyme activity and antioxidant-related molecule content

Drought-treated tartary buckwheat leaves were collected, thoroughly washed with distilled water, and absorbed surface moisture. A 0.1 g sample was weighed and homogenized in 9 times volumes of physiological saline on an ice-water bath to prepare a 10% tissue homogenate. The homogenate was centrifuged at 3,500 rpm for 10 min, and the supernatant was collected for further analysis. The activities of T-SOD, POD, CAT, and GSH-Px enzymes, as well as the contents of GSH, Pro, MDA, and H₂O₂ in tartary buckwheat leaves, were measured using assay kits from Nanjing Jiancheng Bioengineering Institute. Each treatment was replicated three times.

### 2.11 Statistical analysis

The data were processed and analyzed through IBM SPSS 27, while graphs were plotted using Origin 2021. Before analysis, the data were tested for normality and homogeneity of variance. Significant differences among treatments were evaluated by analysis of one-way ANOVA and Tukey’s HSD *post-hoc* test, with a significance threshold of *p* < 0.05. Results are presented as Mean ± SD.

## 3 Results

### 3.1 Evaluation of *Serendipita indica* colonization on the root-associated microbial community of tartary buckwheat

#### 3.1.1 Diversity and richness of the root-associated microbial community in tartary buckwheat

Following a 30-day period of joint cultivation with *S. indica*, the presence of bacteria was noted through the application of trypan blue staining. Findings verified the successful colonization of tartary buckwheat roots by *S. indica* (Supplementary Figure S2). Examining the diversity of microbial communities linked to roots showed that introducing *S. indica* markedly decreased the variety of bacteria in the soil of the rhizospheres (Table 1). Nonetheless, the introduction of *S. indica* did not markedly alter the variety of fungal populations in the soil of the rhizosphere or the endophytic fungi in the roots. The beta diversity was further examined using Jaccard NMDS plots. Our research revealed notable disparities in the composition of rhizosphere soil bacteria (Supplementary Figure S3A) and endophytic fungi communities (Supplementary Figure S3C) between tartary buckwheat treated with *S. indica* and the untreated counterpart.

**Table 1:** Statistical analysis of alpha diversity index.

	Group	ACE	Chao1	Simpson	Shannon
Rhizosphere soil bacteria	CK	1377.35 ± 186.32	1375.92 ± 187.04	0.9968 ± 0.00*	9.3315 ± 0.06
	SI	1135.18 ± 16.87	1132.72 ± 17.07	0.9960 ± 0.00	9.1294 ± 0.04
Rhizosphere soil fungi	CK	523.33 ± 242.67	523.33 ± 242.67	0.9717 ± 0.01	6.859 ± 0.45
	SI	492.00 ± 43.93	492.00 ± 43.93	0.9443 ± 0.01	5.9751 ± 0.21
Endophytic fungi	CK	216.33 ± 13.38	216.33 ± 13.38	0.9373 ± 0.01	4.8163 ± 0.19
	SI	233.00 ± 8.02	233.00 ± 8.02	0.9084 ± 0.01	4.5189 ± 0.09

“*” represent significant differences between different groups (Student’s *t*-test, *p < 0.05; *n* = 3).

#### 3.1.2 *Serendipita indica* inoculation regulates the composition of root-associated microbial community in tartary buckwheat

**Figure 1:**
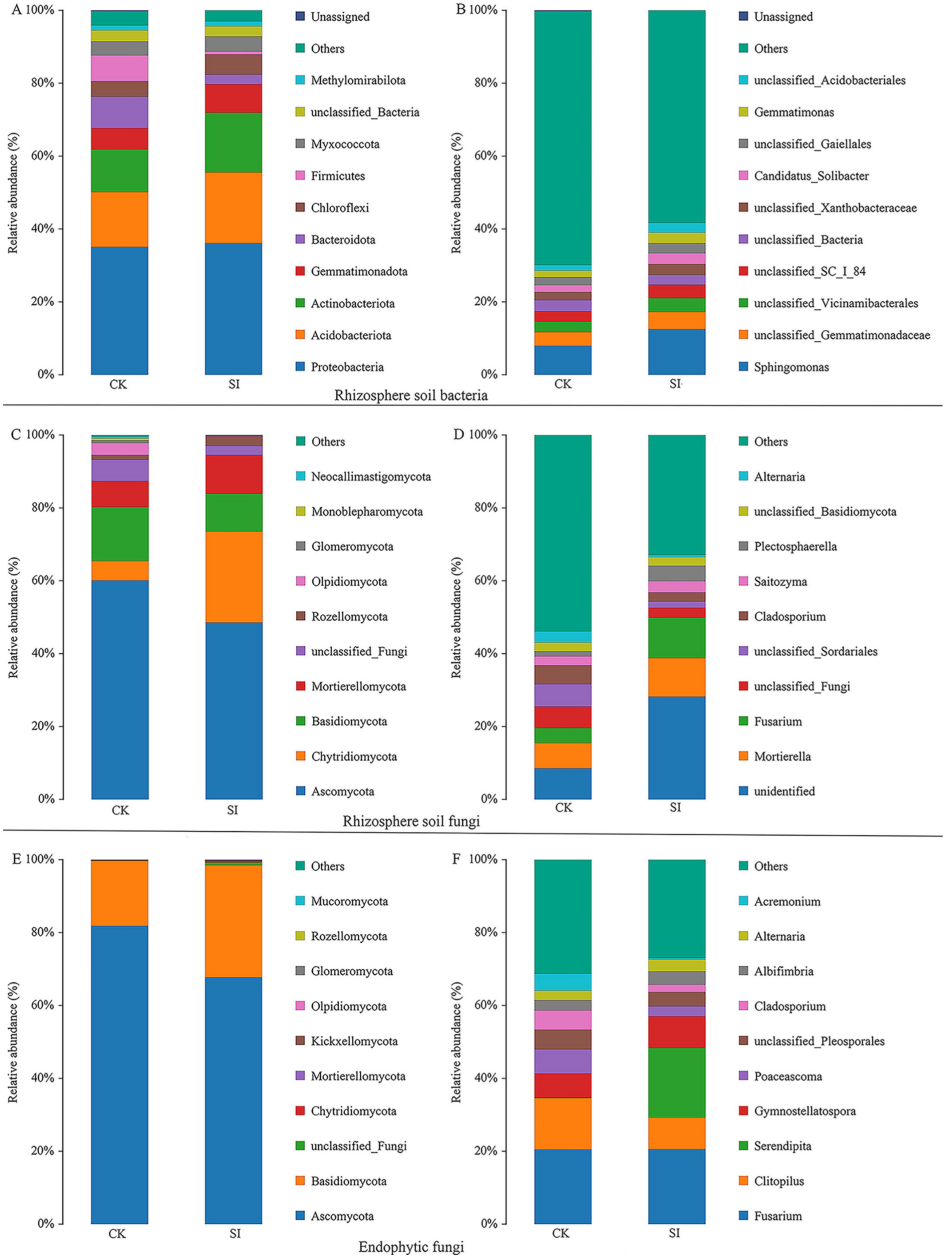
Rhizosphere soil bacterial phyla **(A)** and genera **(B)**; Rhizosphere soil fungal phyla **(C)** and genera **(D)**; Endophytic fungal phyla **(E)** and genera **(F)**.

Notable variations were observed in the composition of microbial communities associated with the roots of tartary buckwheat treated with *S. indica* and in the control group without inoculation. Inoculation with *S. indica* led to an increase in the prevalence of Acidobacteriota, Actinobacteriota, Gemmatimonadota, and Chloroflexota, but simultaneously decreased the presence of Bacteroidota and Firmicutes at the bacterial phylum level (Figure 1A). Within the fungal phylum, inoculation with *S. indica* led to an increase in the prevalence of Chytridiomycota, Mortierellomycota, and Rozellomycota, but a reduction in the numbers of Basidiomycota and Olpidiomycota (Figures 1C,E).

**Figure 2:**
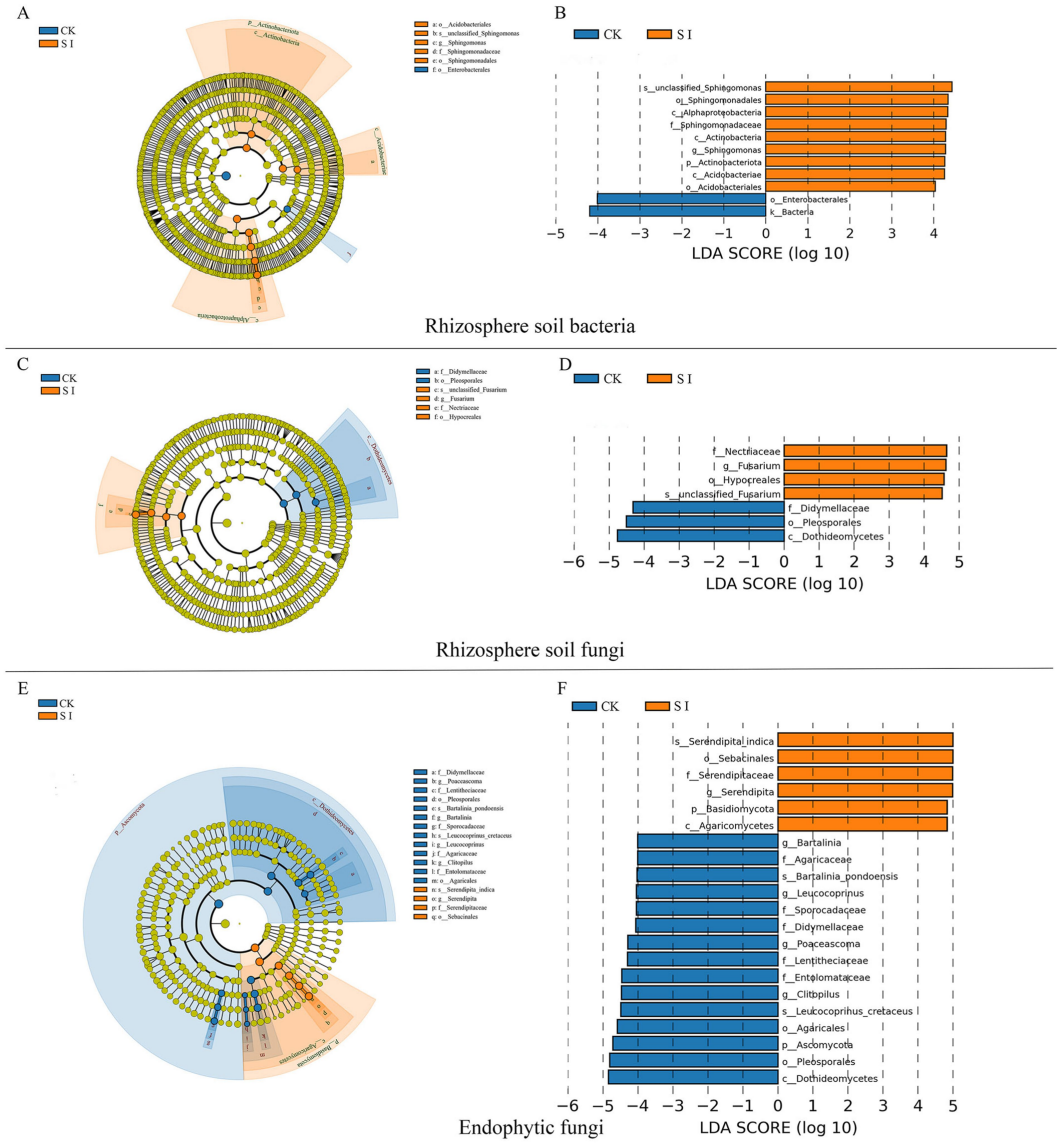
Rhizosphere soil bacterial LEfSe analysis cladogram diagram **(A)** and LDA score distribution histogram **(B)**; Rhizosphere soil fungal LEfSe analysis cladogram diagram **(C)** and LDA score distribution histogram **(D)**; Endophytic fungal LEfSe analysis cladogram diagram **(E)** and LDA score distribution histogram **(F)**.

Additionally, our attention was on bacterial and fungal genera capable of enhancing plant growth, such as *Sphingomonas*, *Gemmatimonas*, *Mortierella*, and *Fusarium*, which exhibited notable differences between the groups with and without *S. indica* inoculation. Inoculation with *S. indica* notably boosted the prevalence of *Sphingomonas*, *Candidatus_Solibacter*, *Gemmatimonas*, *Mortierella*, *Fusarium*, and *Plectosphaerella*, but diminished the presence of *Cladosporium*, *Alternaria*, and *Acremonium* (Figures 1B,D,F). The LefSe study showed that inoculating *S. indica* markedly enhanced the presence of eight bacterial groups, namely Actinobacteriota, Actinobacteria, Alphaproteobacteria, Acidobacteriae, Acidobacteriales, Sphingomonadales, Sphingomonadaceae, and *Sphingomonas*. Furthermore, there was a notable increase in five fungal groups, namely Basidiomycota, Agaricomycetes, Hypocreales, Nectriaceae, and *Fusarium* (Figure 2).

### 3.2 Determination of *in vitro* plant growth-promoting traits of strains and construction of composite microbial consortia

#### 3.2.1 *In vitro* plant growth-promoting traits of strains

Following the analysis of microbial diversity, we chose six strains from 16 bacterial and 48 fungal strains, known for their potential synergistic effects with *S. indica*, which were previously isolated in our lab. Subsequently, the growth-enhancing characteristics of these selected strains *in vitro* were assessed (Table 2). The findings revealed that, out of the seven strains examined, six, with the exception of JQB3, could dissolve inorganic phosphate, reaching a peak concentration of 152.92 mg/L in JQF1. Each strain demonstrated potent capabilities in dissolving potassium, with soluble potassium levels in the culture medium varying between 192.48 and 895.19 mg/L. IAA plays a crucial role in fostering plant development, and among the strains examined, five could produce IAA, with *S. indica* achieving the peak concentration of 50.83 mg/L. Results from the siderophore production test indicated the ability of three strains to produce and emit siderophores, resulting in the formation of orange-yellow halos surrounding their colonies. The D/d ratio, representing the ratio of yellow halo diameter to colony diameter, varied between 1.53 and 2.03. Furthermore, every strain, except for JQB2 and JQF1, showed the capability to fix nitrogen. A quartet of bacterial strains thrived in an environment solely supplied with ACC nitrogen, signifying their capacity to suppress ethylene production and boost the plant’s resilience to abiotic environmental stress.

**Table 2:** The results of *in vitro* plant growth-promoting traits of strains.

Strains	Species	Available P (mg/L)	Soluble K (mg/L)	IAA content (mg/L)	Siderophores (D/d)	Nitrogen fixation	ACC deaminase activity
JQB1	*Bacillus cereus*	54.86 ± 1.37	895.19 ± 11.61	10.40 ± 0.27	1.53	+	+
JQB2	*Rhodobacter sphaeroides*	5.69 ± 2.60	773.39 ± 1.54	1.11 ± 0.12	−	−	+
JQB3	*Rhodococcus sphaeroides*	−	339.22 ± 4.43	7.03 ± 0.48	1.83	+	+
JQF1	*Serendipita indica*	152.92 ± 2.10	499.24 ± 5.31	50.83 ± 2.48	−	−	+
JQF2	*Mortierella alpina*	67.36 ± 0.43	374.49 ± 5.80	−	−	+	−
JQF3	*Ceriporia lacerata*	7.53 ± 0.49	192.48 ± 3.07	4.30 ± 0.16	2.03	+	−
JQF4	*Fusarium equiseti*	19.19 ± 0.43	390.34 ± 4.60	−	−	+	−

“−” means that the strain does not have this characteristic, “+” means the strain has this characteristic; D/d means yellow halo diameter/colony diameter.

#### 3.2.2 Compatibility among different strains

**Figure 3:**
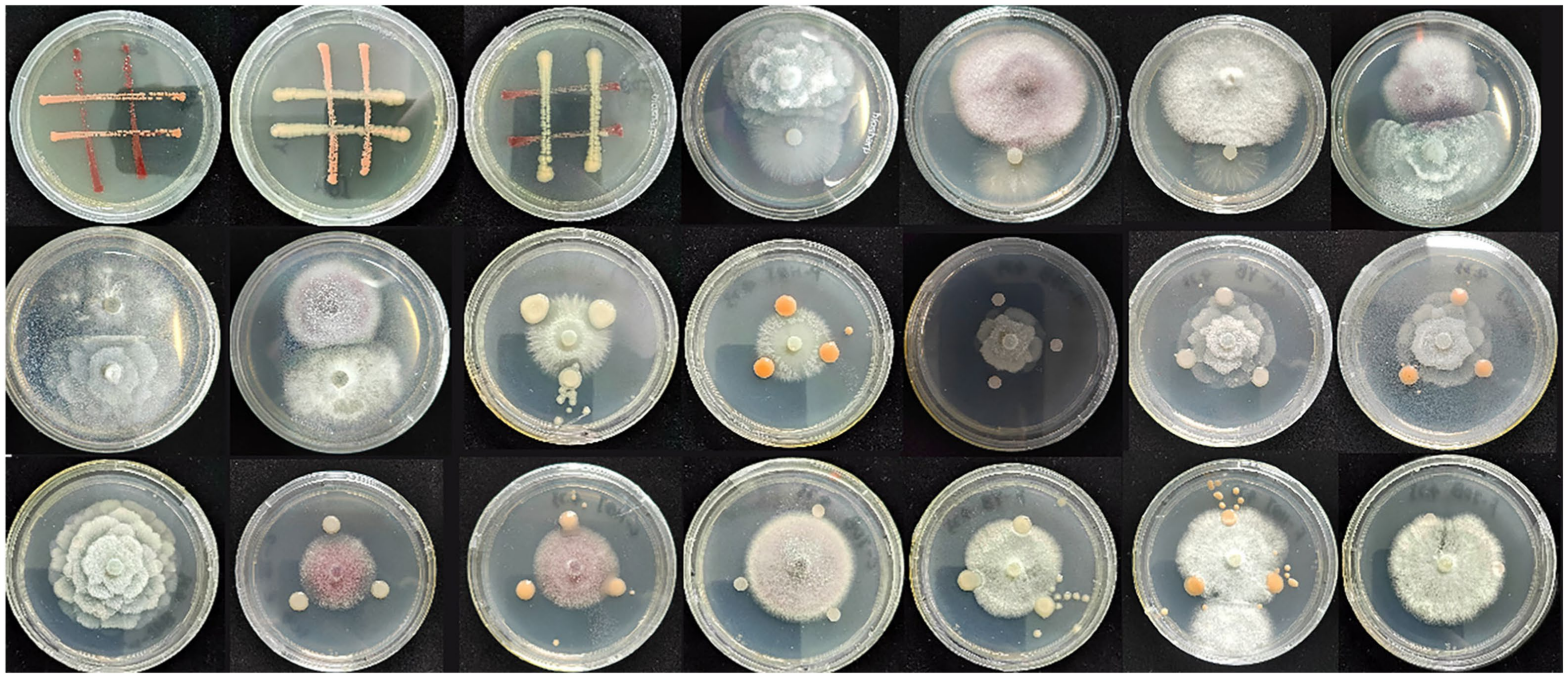
The results of compatibility assays among different strains.

The results of the compatibility assays among all strains are presented in Figure 3 and Supplementary Table S1. An inhibitory effect was observed between the JQB1 and JQB2 strains, with a notable reduction in JQB2 growth at the junction. Moreover, separate zones of inhibition were noted between strains JQF2 and JQF3, as well as between JQF3 and JQF4, suggesting reciprocal inhibitory effects. All fungal strains suppressed the proliferation of strain JQB2, and reciprocal inhibition was observed between JQB3 and JQF3. Given the inhibition of JQB2 by all strains except JQB3, the selection of strains JQB1, JQB3, JQF1, JQF2, JQF3, and JQF4 was made for the creation of composite microbial groups. Supplementary Table S2 displays the outcomes for each combination of strains.

### 3.3 Effects of the composite microbial consortia on growth parameters and photosynthesis of tartary buckwheat

#### 3.3.1 Effects of the composite microbial consortia on the plant growth phenotypes

**Figure 4:**
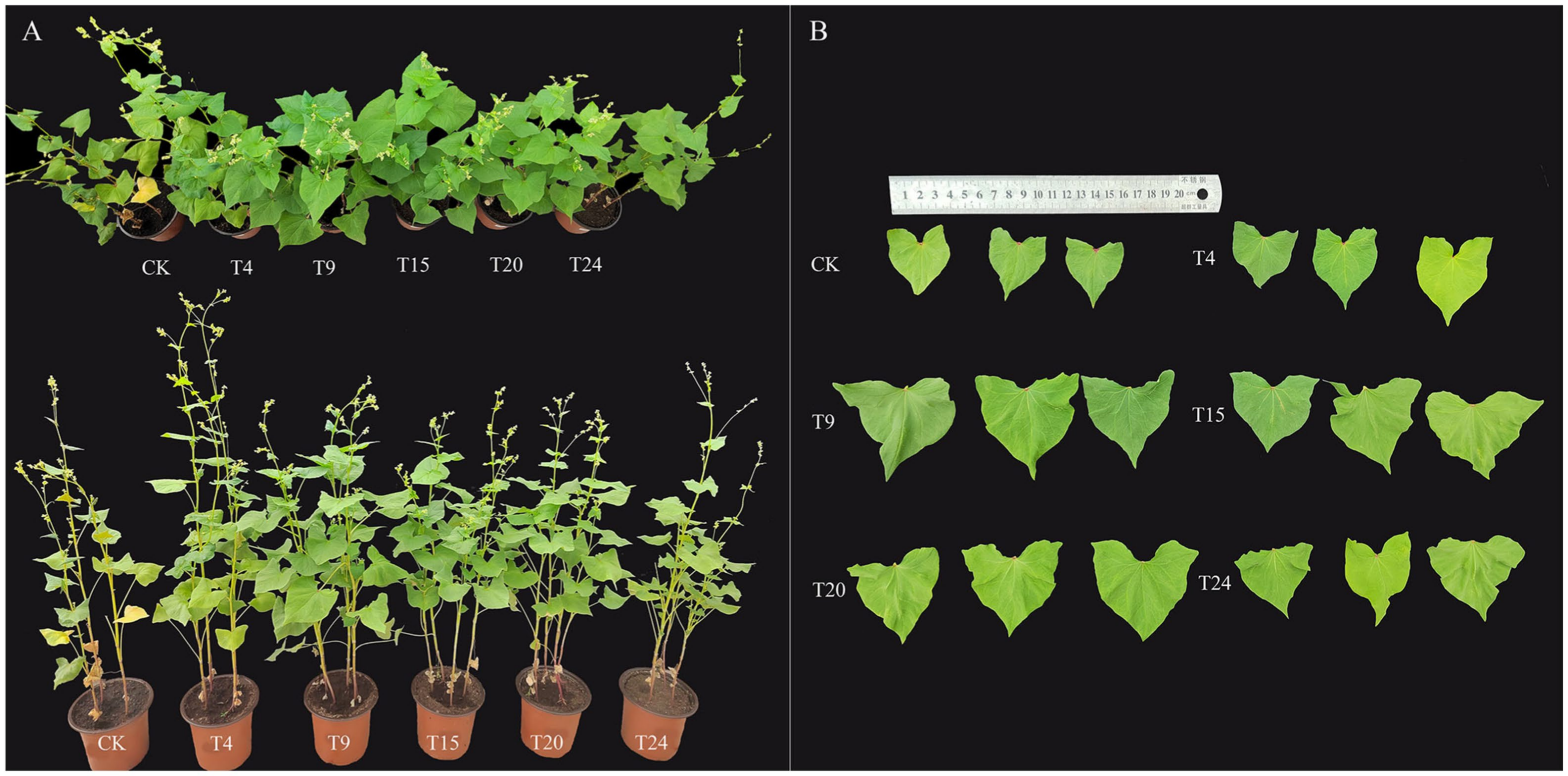
Comparison of tartary buckwheat **(A)** plant phenotypes and **(B)** leaf size at the 7th node in different treatment groups.

**Figure 5:**
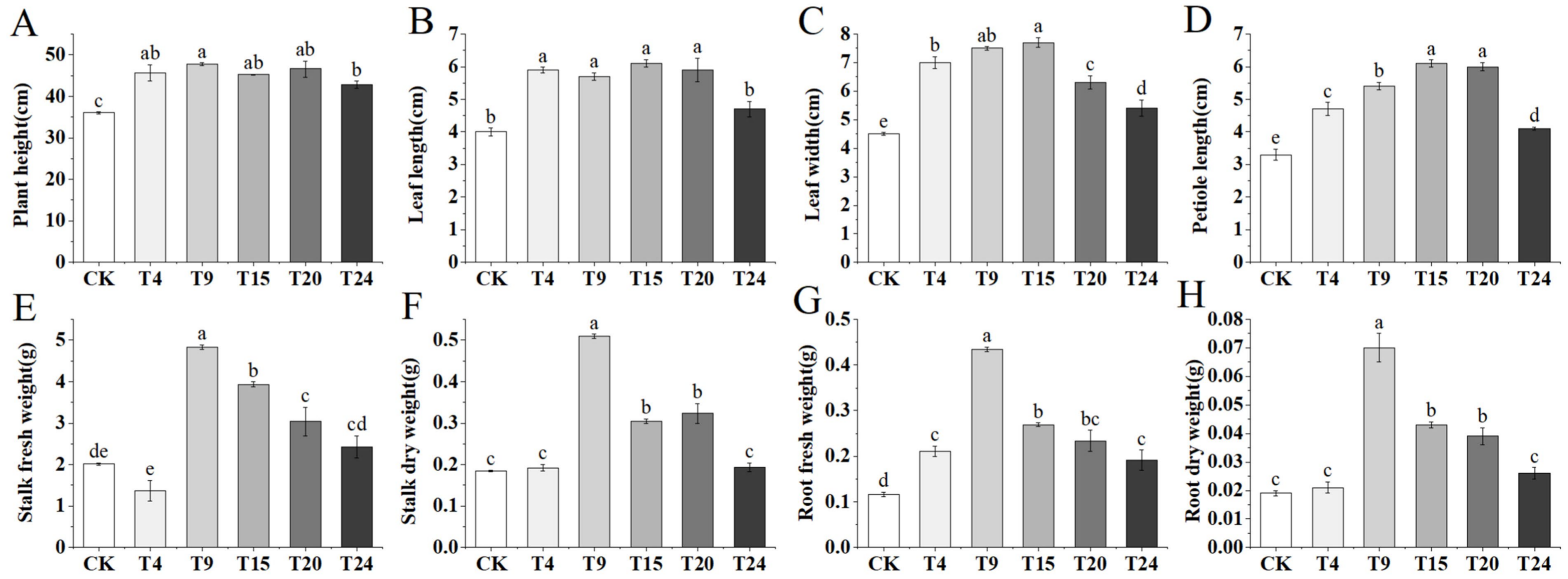
Growth-related parameters of tartary buckwheat in different treatment groups: **(A)** Plant height; **(B)** Leaf length; **(C)** Leaf width; **(D)** Petiole length; **(E)** Stalk fresh weight; **(F)** Stalk dry weight; **(G)** Root fresh weight; **(H)** Root dry weight. Different letters indicate significant differences (*n* = 10, *p* < 0.05).

Following the results of the strain compatibility tests, 24 different combinations of microbial consortia were developed. Supplementary Table S3 displays the impact of various mixtures on the growth metrics of tartary buckwheat. From each combination of strain numbers, the top-performing group was selected for further experimental and analytical work. Relative to the control group, inoculation solely with *S. indica* and microbial groups significantly enhanced tartary buckwheat growth, leading to a notable increase in plant stature, leaf size, and reduced leaf discoloration (Figure 4). In contrast to the control group, the T9 microbial group enhanced tartary buckwheat’s height, leaf length, width, and petiole length by 32.7, 42.5, 66.6, and 63.6%, respectively (Figures 5A–D). Additionally, tartary buckwheat’s leaf width and petiole length in the T9 and T15 groups notably exceeded those in the T4 group, which received only *S. indica* inoculation. Furthermore, the microbial groups T9, T15, and T20 markedly enhanced both the fresh and dry mass of stems, as well as the fresh and dry mass of roots in tartary buckwheat. Within this group, the T9 group showed the most significant growth, with increases in fresh stem weight, dry stem weight, fresh root weight, and dry root weight of 139.6, 175.1, 274.1, and 250% respectively, compared to the control (Figures 5E–H). Despite the T4 and T24 groups encouraging an increase in fresh root mass, their impact on the weight of fresh stems, dry stems, or dry roots was not significant.

#### 3.3.2 Effects of the composite microbial consortia on photosynthesis in tartary buckwheat

**Figure 6:**
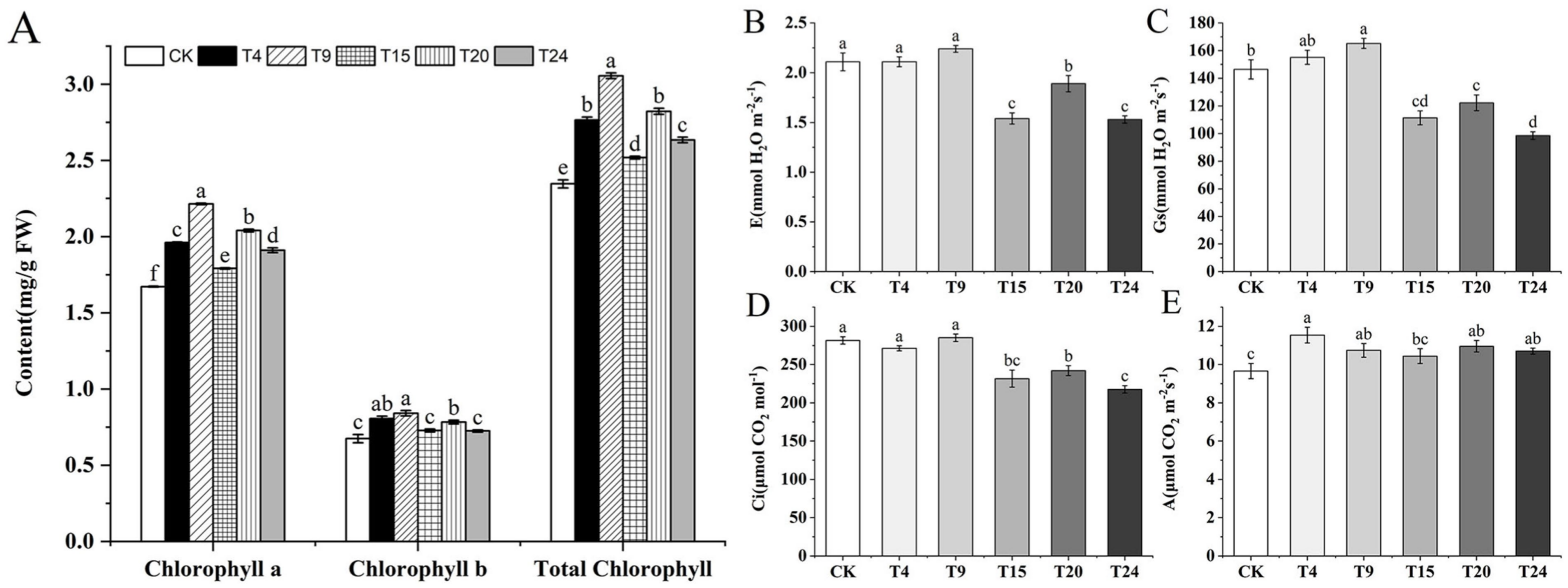
**(A)** Chlorophyll content of tartary buckwheat leaves in different treatment groups; Photosynthetic parameters of tartary buckwheat leaves: **(B)** Transpiration rate (E); **(C)** Stomatal conductance (Gs); **(D)** Intercellular CO_2_ concentration (CI); **(E)** Net photosynthetic rate (A). Different letters indicate significant differences (*n* = 10, *p* < 0.05).

Photosynthesis is a crucial process in plant development, serving as the primary energy source for both plant growth and nutrient acquisition. The efficiency and intensity of photosynthesis in plants are directly affected by their chlorophyll levels. Consequently, the chlorophyll levels and photosynthetic characteristics of tartary buckwheat leaves were assessed in each treatment group (Figure 6). Every treatment group exhibited a notable rise in the levels of total chlorophyll, chlorophyll a, and chlorophyll b in tartary buckwheat leaves, with the T9 group demonstrating the most substantial increase. Relative to the control group, the T9 group’s total chlorophyll, chlorophyll a, and chlorophyll b levels increased by 30.2, 32.5, and 24.6%, respectively. Furthermore, the status of stomata, whether open or closed, is crucial in affecting plant photosynthesis. Relative to the control group, the T4 and T9 groups significantly enhanced the stomatal conductance in tartary buckwheat leaves, but they did not significantly influence the rate of transpiration or the concentration of CO_2_ between cells. In contrast, the rates of transpiration, stomatal conductance, and intercellular CO_2_ levels were significantly reduced in the T15, T20, and T24 treatment groups compared to the control group. In every treatment group except T15, buckwheat leaves exhibited a notably greater net photosynthetic rate than the control group, with the T9 group experiencing the most substantial 19.3% increase compared to the control group.

### 3.4 Effects of the composite microbial consortia on soil physicochemical properties and enzyme activities in tartary buckwheat

#### 3.4.1 Effects of the composite microbial consortia on soil structure

**Figure 7:**
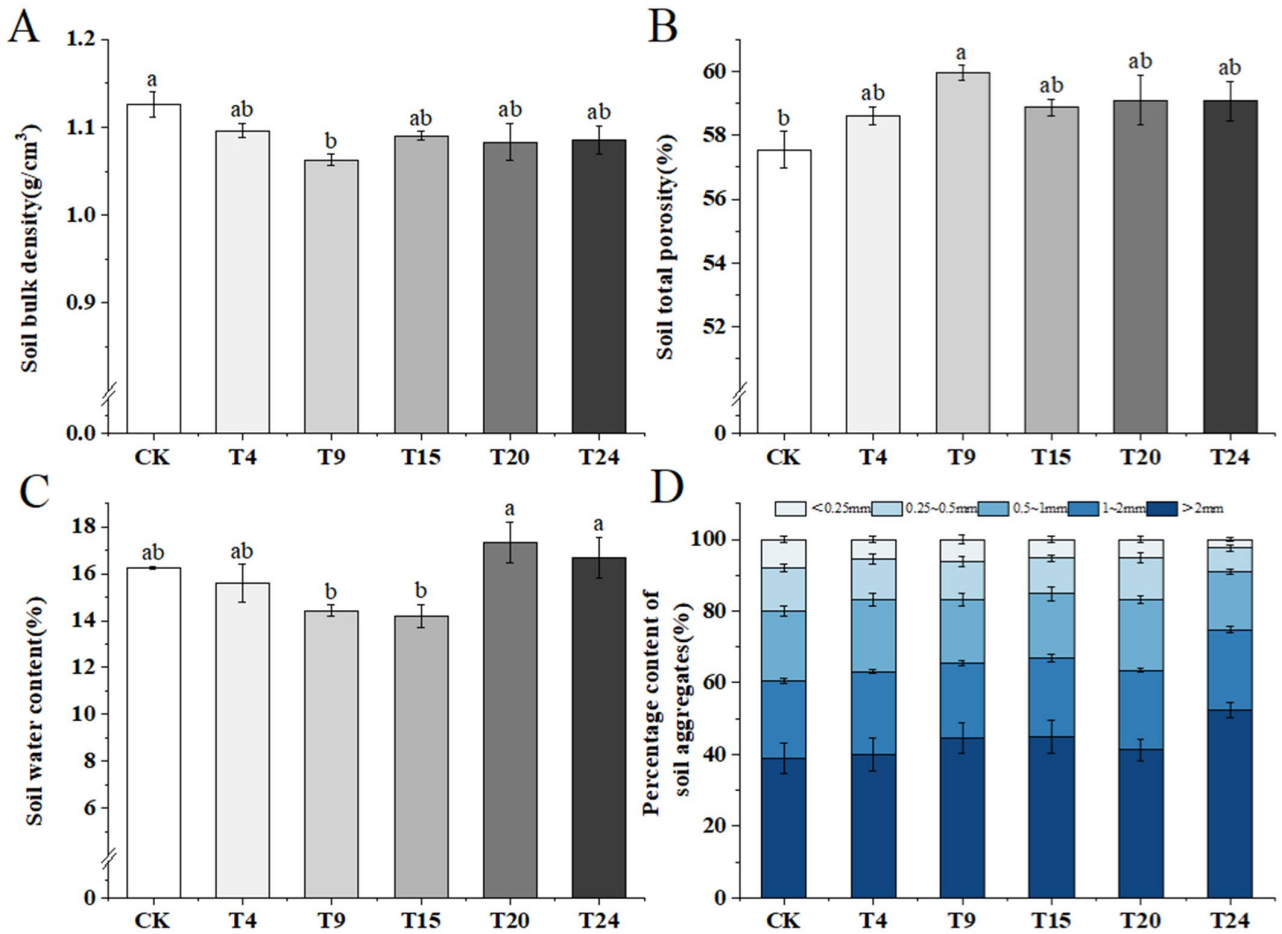
Comparison of physical properties of tartary buckwheat soils in different treatment groups: **(A)** Soil bulk density; **(B)** Soil total porosity; **(C)** Soil water content; **(D)** Percentage content of soil aggregates. Different letters indicate significant differences (*n* = 3, *p* < 0.05).

Microorganisms are vital in enhancing soil composition through diverse metabolic processes, including the release of extracellular polymers and synergistic interactions with plant roots. Such mechanisms increase the porosity of the soil, promote the formation of more stable macroaggregates, and play a significant role in the overall improvement of the soil’s structure (Jeewani et al., 2021; Hu et al., 2024). Our research focused on examining how various inoculation methods affect the bulk density, overall porosity, moisture levels, and the distribution of soil aggregates in tartary buckwheat. Our findings indicate a notable 6.1% reduction in soil bulk density in the T9 group compared to the control, along with a 4.1% increase in overall soil porosity. There were no notable variances detected between the control and other treatment groups (Figures 7A,B). Soil moisture levels in each treatment group varied between 14.19 and 17.35%, showing no notable variance from the control group (Figure 7C). The development and stability of soil clusters play crucial roles in defining the health and efficiency of the soil. The findings from our experiments show that inoculation enhances the quantity of substantial soil clusters (d > 2 mm) and reduces the number of microaggregates (d < 0.25 mm). Such alterations improve soil aeration and nutrient retention (Figure 7D).

#### 3.4.2 Effects of the composite microbial consortia on the biochemical properties of tartary buckwheat soil

**Figure 8:**
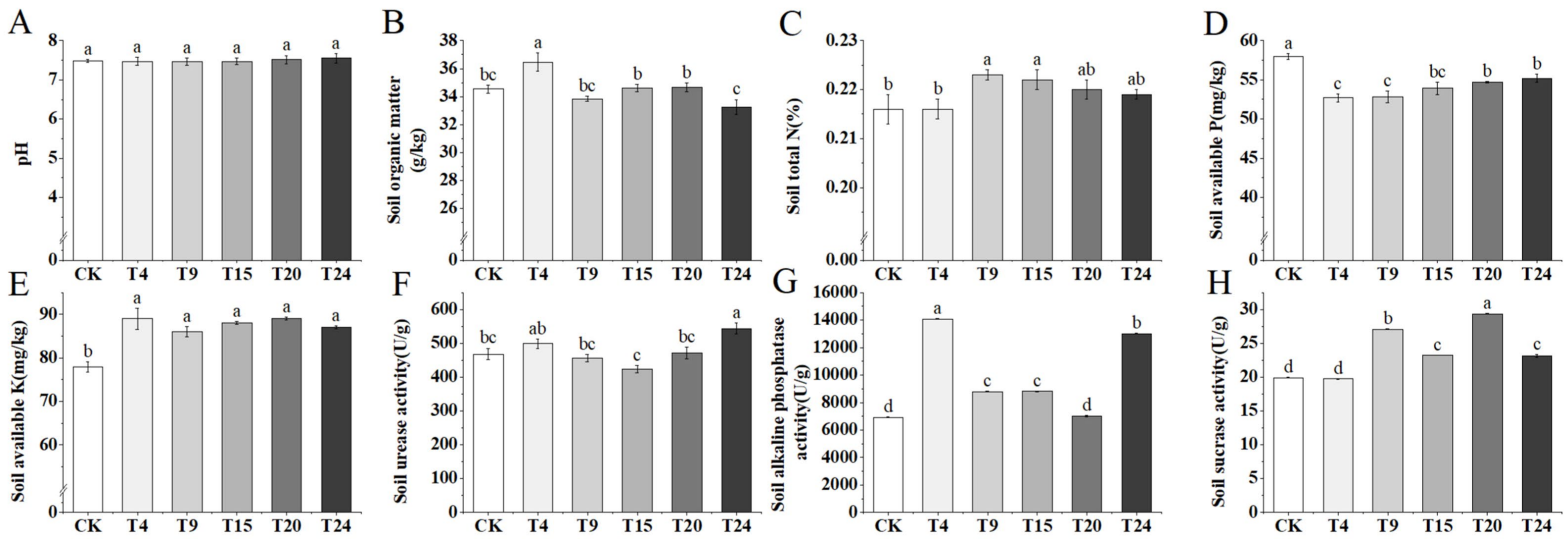
Comparison of soil chemical properties and enzyme activities of tartary buckwheat soils in different treatment groups: **(A)** pH; **(B)** Soil organic matter; **(C)** Soil total N; **(D)** Soil available P; **(E)** Soil available K; **(F)** S-UE activity; **(G)** S-AKP/ALP activity; **(H)** S-SC activity. Different letters indicate significant differences (*n* = 3, *p* < 0.05).

Microorganisms play a pivotal role in promoting soil fertility. Through mechanisms like the breakdown of organic substances, solubilization of P and K, and nitrogen fixation, they facilitate the recycling of nutrients in the soil and are crucial for preserving its health (Sathya et al., 2016). Inoculation treatments had no significant effect on soil pH (Figure 8A). Treatments involving inoculation did not significantly alter the soil’s pH level (Figure 8A). Relative to the control group, there was a notable 5.7% increase in soil organic matter in the T4 group, and a significant 3.7 and 3.2% increase in total nitrogen levels in the T9 and T15 groups, respectively (Figures 8B,C). There was a notable rise in the amount of potassium present in the soil among all the treatment groups, yet there was a reduction in the phosphorus content relative to the control group (Figures 8D,E). The activity of enzymes in the soil serves as an additional vital measure for evaluating soil fertility. Additionally, we assessed the levels of soil urease, alkaline phosphatase, and sucrase in various treatment categories (Figures 8F–H). The findings indicated a notable increase in soil urease activity in the T24 group. With the sole exception of the T20 group, every treatment group demonstrated a significantly elevated level of soil alkaline phosphatase activity compared to the control. Furthermore, except for the T9 group, all other treatment groups exhibited higher soil sucrase activity than the control group.

### 3.5 Effects of the composite microbial consortia on the growth of tartary buckwheat under drought stress

#### 3.5.1 Effects of the composite microbial consortia on stomatal density and root growth in tartary buckwheat

**Figure 9:**
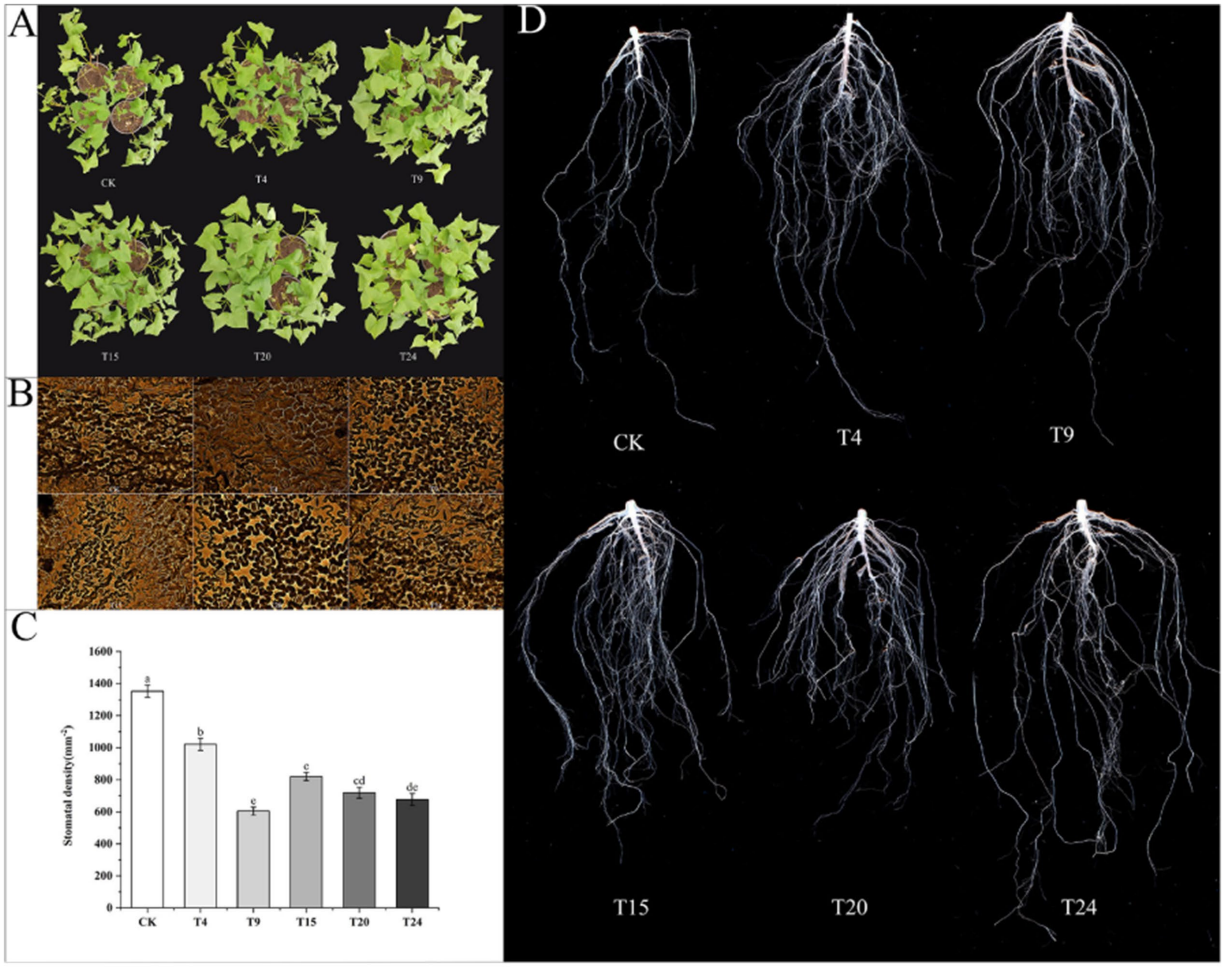
Comparison of buckwheat phenotype **(A)**, stomatal opening/closing status **(B)**, stomatal density **(C)** and root morphology **(D)** in different treatment groups under drought conditions. Different letters indicate significant differences (*n* = 3, *p* < 0.05).

After enduring a three-day spell of natural drought, the control group of tartary buckwheat exhibited heightened symptoms of wilting and a lack of water (Figure 9A). Research indicates that plant growth-enhancing microorganisms can strengthen drought resilience in plants through multiple methods, such as managing stomatal conditions (Mishra et al., 2020) and promoting root system development (Li et al., 2023a), thereby improving water retention and absorption in drought conditions. Observing the stomata in tartary buckwheat leaves across different treatment groups showed a significantly elevated stomata count in the control group relative to other treatment groups (Figures 9B,C). In times of drought, plants have the potential to substantially lower water loss through transpiration by reducing openings in their stomata. Further analysis of root architecture under drought conditions revealed that the root systems of tartary buckwheat in the inoculated groups were significantly more developed (Figure 9D). In this cohort, the T4 group exhibited the highest number of root tips, exceeding the control group’s count by a multiple of 6.26. Compared to the control group, the T9 group showed the highest average root size and volume, with measurements 1.11 and 3.35 times greater, respectively. Additionally, the T15 group showed the greatest root length and surface area, with measurements 3.44 and 3.43 times higher than those of the control group, respectively (Table 3).

**Table 3:** Comparison of root growth parameters in tartary buckwheat under drought stress among different treatment groups.

Treatment	Root length (mm)	Average root diameter (mm)	Root volume (mm^3^)	Root surface area (mm^2^)	Number of root tips (count)
CK	463.1^e^	0.17^c^	21.4^d^	240.61^d^	164^d^
T4	1476.4^b^	0.16^c^	55.4^c^	752.10^b^	1027^a^
T9	1233.6^cd^	0.19^a^	71.9^a^	745.10^b^	634^c^
T15	1595.6^a^	0.17^c^	64.6^b^	827.44^a^	912^b^
T20	1197.7^d^	0.18^ab^	61.8^b^	690.29^c^	570^c^
T24	1326.5^c^	0.17^b^	64.5^b^	734.47^bc^	613^c^

Different letters indicate significant differences (*n* = 3, *p* < 0.05).

#### 3.5.2 Effects of the composite microbial consortia on the antioxidant system in tartary buckwheat leaves under drought stress

**Figure 10:**
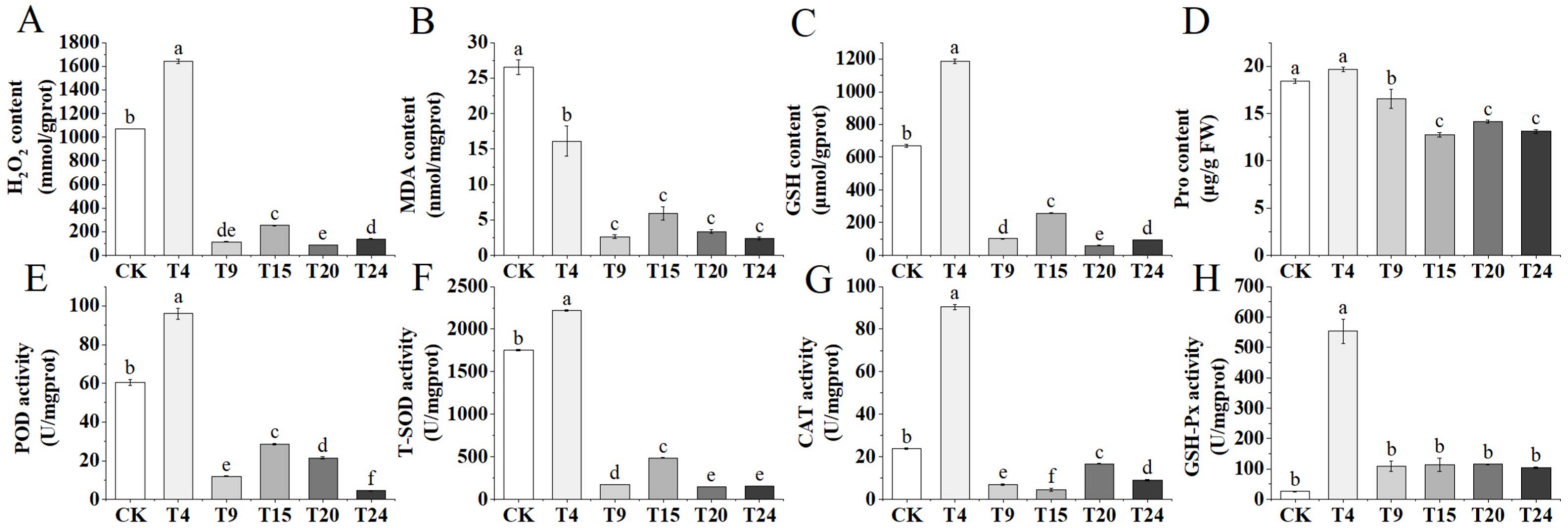
Changes in antioxidant enzyme activities and stress-related molecule levels in tartary buckwheat leaves under drought stress among different treatment groups: **(A)** H_2_O_2_ content; **(B)** MDA content; **(C)** GSH content; **(D)** Pro content; **(E)** POD activity; **(F)** T-SOD activity; **(G)** CAT activity; **(H)** GSH-Px activity. Different letters indicate significant differences (*n* = 3, *p* < 0.05).

Our additional analysis focused on the concentrations of various antioxidant enzymes and molecules in tartary buckwheat leaves across different treatment groups experiencing drought stress. Plants frequently encounter elevated levels of reactive oxygen species (ROS) in their cells during drought conditions (Li et al., 2022). The findings indicated a notable increase in H_2_O_2_ and MDA levels in tartary buckwheat leaves in both the control and *S. indica*-only inoculated groups, in contrast to the other microbial consortia groups (Figures 10A,B). Furthermore, the levels of GSH and proline in the foliage of both the control and *S. indica*-treated groups surpassed those in the other groups (Figures 10C,D). The findings reveal that tartary buckwheat leaf cells in both the control and T4 groups experienced increased oxidative stress and cellular damage. Additionally, the activities of POD, T-SOD, and CAT enzymes in the leaves of tartary buckwheat were significantly elevated in both the control and T4 treatment groups compared to those in the other groups. In the T4 group, the activity of GSH-Px was markedly greater than that of the control group; however, there were no notable differences between the other treatment groups and the control group (Figures 10E–H).

## 4 Discussion

The development of plants is intimately linked to microorganisms associated with roots, encompassing both rhizosphere and endophytic microorganisms (Bai et al., 2022). A multitude of elements, such as the nature of the soil, the growth phase of plants, and the interplay between microorganisms, shape the composition of root microbial communities (Peiffer et al., 2013; Shakya et al., 2013; Schreiter et al., 2014; Zarraonaindia et al., 2015; Musyoki et al., 2016; Chaudhary et al., 2020). Research indicates that close to 86% of external microbial inoculations may impact indigenous root microbial populations, either briefly or over an extended period (Mawarda et al., 2020; Sui et al., 2024). Research indicates that *S. indica* may boost the presence of bacteria resistant to arsenic, such as *Lysobacter* and *Steroidobacter*, in the rhizosphere of *Artemisia annua*, enhancing the plant’s resistance to arsenic stress (Rahman et al., 2023). Our research revealed that introducing *S. indica* markedly decreased the variety of bacteria in the rhizosphere soil of tartary buckwheat, possibly due to the competitive dynamics between *S. indica* and the indigenous bacterial populations in the rhizosphere (Barahona et al., 2011; Innerebner et al., 2011). Studies have shown that specific fungi have the ability to emit substances that either inhibit or stimulate growth through their mycelia, impacting the development of specific rhizosphere bacteria. Furthermore, *S. indica* has the potential to modify the makeup of rhizosphere bacteria indirectly through alterations in root exudation patterns (Akyol et al., 2019). Furthermore, NMDS studies showed that introducing *S. indica* markedly changed the makeup of rhizosphere bacteria and endophytic fungi communities, aligning with earlier research indicating that microbial modifications can transform the rhizosphere microbiome (Saravanakumar et al., 2017; Huang et al., 2021). Administering *S. indica* led to a rise in Acidobacteriota, Actinobacteriota, Gemmatimonadota, and Chloroflexota populations, many of which are known to be vital in enhancing plant development and the cycling of nutrients in soil (Shen et al., 2015; Jog et al., 2016; Lin et al., 2018). When integrated with LEfSe analysis, our findings indicate that introducing *S. indica* into the soil enhances the presence of advantageous microbes like *Sphingomonas*, *Gemmatimonas*, and *Mortierella*. The widespread presence of *Sphingomonas* and *Gemmatimonas* in soil has been identified as crucial for enhancing plant growth and aiding in soil restoration (Asaf et al., 2020; Xu et al., 2023). It has been documented that *Mortierella* produces IAA, GA, and ACC deaminase, which boosts winter wheat growth (Ozimek et al., 2018) and plays a role in soil phosphorus conversio (Wang Y. et al., 2022). Fascinatingly, it was noted that the inoculation led to a rise in *Fusarium* populations, a genus widely recognized for causing root rot diseases in various plants, including wheat root rot (Li et al., 2023b) and maize stalk rot (Liu et al., 2022). Recent research indicates that specific species of *Fusarium* may have the capability to enhance plant growth (Feng et al., 2023). Our research found no evidence of root rot infection in tartary buckwheat, prompting us to theorize that the *Fusarium* species, which was enriched post-inoculation with *S. indica*, is likely non-pathogenic. Additionally, administering *S. indica* led to a decrease in the prevalence of harmful genera such as *Cladosporium*, *Alternaria*, and *Acremonium*. A significant number of these genera have been identified as being linked to plant ailments, including leaf spot and wilt (Hou et al., 2019; Mukhtar et al., 2020; Gou et al., 2022). This implies that *S. indica* could be a promising subject for further investigation to improve resistance to plant diseases.

Microorganisms linked to roots enhance plant development through multiple pathways, such as producing phytohormones, dissolving phosphates, stabilizing nitrogen, and generating siderophores (Olanrewaju et al., 2017; Elsherbiny et al., 2023). Our assessment focused on the growth-enhancing characteristics of the seven selected strains for creating microbial consortia (Table 2). The findings revealed that each strain exhibited at least two traits that enhance plant growth, highlighting its significant capacity to promote plant development. Plant growth heavily depends on phosphorus and potassium, with root-associated microbes assisting in their availability by dissolving insoluble phosphates and potassium salts in the soil, thereby transforming them into more plant-absorbable forms (Muthuraja and Muthukumar, 2022; Wang Z. et al., 2022). Furthermore, through the production of siderophores and nitrogen fixation, microorganisms can boost plants’ absorption of iron and nitrogen, fulfilling their essential nutrient needs (Radzki et al., 2013). Plants primarily obtain water and nutrients from the soil via roots. Microorganisms linked to roots are capable of directly generating or triggering the creation of IAA in plants (Zhao, 2010; Saini et al., 2013; Mehmood et al., 2019; Chen et al., 2021), thus aiding in root growth and boosting the absorption of water and nutrients (Khoso et al., 2024). A study by Lin et al. (2022) found that the combined application of multiple bacteria with IAA-producing capabilities significantly promotes root growth in *Astragalus mongholicus* under drought stress. Likewise, our research indicated that inoculating solely with *S. indica* and various microbial groups markedly enhanced the development of tartary buckwheat roots (Figure 9D), the rise in root length, volume, surface area, and the number of root tips (Table 3). Leaves, key locations for photosynthesis, function as the plant’s “production sites” (Kohli et al., 2020). Results from pot tests revealed that tartary buckwheat, when treated with *S. indica* or its microbial groups, had larger leaf sizes and reduced leaf discoloration compared to the control (Figure 5). The expanded area of leaves allows plants to harness more light energy for photosynthesis. Additionally, the inoculation markedly increased the chlorophyll levels in buckwheat leaves, with the T9 and T20 groups exhibiting higher chlorophyll concentrations compared to the sole inoculation of *S. indica*. This phenomenon could be attributed to the siderophore-producing bacteria in these groups, which produce siderophores to enhance plant iron absorption, thus facilitating chlorophyll synthesis (Yue et al., 2022).

The soil is crucial for plant growth, affecting root development and the uptake of nutrients. Microorganisms, being a crucial part of the soil, play a key role not only in its structural composition but also in significantly influencing the biogeochemical processes of carbon, nitrogen, phosphorus, and various other nutrients (Sathya et al., 2016). The composition of soil, including its aggregates and porosity, among other factors, influences the circulation of water, oxygen, and nutrients within the soil. The overall density and porosity of soil are widely regarded as key metrics for assessing its quality. Our research revealed that the T9 treatment group significantly decreased the bulk density of soil and increased its porosity, thus improving its ability to retain water and nutrients, fostering an optimal environment for root growth and development. Furthermore, our findings indicate that inoculation with microbial consortia led to an increase in the concentration of substantial soil aggregates (>2 mm) in tartary buckwheat soil. Earlier research has shown that larger aggregates, in contrast to microaggregates (less than 0.25 mm), have higher concentrations of organic carbon, nitrogen, and particulate organic matter (Beare et al., 1994; Plante and McGill, 2002). A rise in substantial aggregate levels will boost the organic carbon nutrients in the soil and enhance the circulation of water and air in the soil (Olayemi et al., 2022). Organic matter in soil plays a crucial role in the composition of soil organic carbon and is vital for the fertility of the soil and the cycling of ecosystems (Zhang et al., 2021; Wei et al., 2024). Research has shown that using *Pseudomonas chlororaphi* and *Bacillus altitudinal* together boosts organic carbon and potassium levels in soybean soil, simultaneously fostering soybean development and increasing crop yield (Zhang et al., 2023). The findings of our research revealed that introducing *S. indica* into the soil of tartary buckwheat enhanced its organic content. Furthermore, the introduction of *S. indica* and its microbial groups markedly increased the soil’s potassium levels, possibly due to the strains ability to dissolve insoluble potassium salts in the soil (Basak and Biswas, 2010). A reduction in the phosphorus content of the soil could be attributed to the improved absorption and use of phosphorus by various microorganisms and plants (Richardson et al., 2011; Mansotra et al., 2015). The enhanced absorption of phosphorus by plants could result in fewer phosphate-accumulating bacteria, thus affecting the diversity of bacterial populations in the rhizosphere soil (Rodríguez-Caballero et al., 2017). Enzymes in the soil are crucial for controlling nutrient recycling and the breakdown and mineralization of organic substances, with their functions mirroring the soil’s carbon cycling ability, properties, and fertility rates (Song et al., 2021). Our research extended to examining how inoculation impacts the functions of urease, alkaline phosphatase, and sucrase in the soil of tartary buckwheat. The results indicated a notable rise in soil urease activity within the T24 treatment cohort. Furthermore, introducing *S. indica* and its microbial groups typically boosts soil alkaline phosphatase and sucrase levels, thus facilitating the movement of carbon, nitrogen, and phosphorus in the soil and enhancing its nutritional value.

Drought, in its natural form, is regarded as the gravest non-living element that restricts the expansion and production of diverse crops worldwide. The absence of water directly hinders the absorption of nutrients and diminishes the photosynthetic processes in plants (Farooq et al., 2012). Growing research indicates that microorganisms linked to roots may mitigate stress-related harm in plants by synthesizing indole-3-acetic acid (IAA), 1-aminocyclopropane-1-carboxylate (ACC) deaminase, and extracellular polysaccharides (Khan and Singh, 2021). Plants frequently overproduce ethylene under non-living stressors, hindering the growth of roots and shoots. Some microorganisms linked to roots are capable of synthesizing ACC deaminase, leading to the breakdown of ACC, ethylene’s forerunner, thus diminishing plant ethylene concentrations and mitigating its suppressive impacts (Gamalero and Glick, 2015; Singh et al., 2015). Our research pinpointed four variants capable of synthesizing ACC deaminase, successfully curbing the overproduction of ethylene in tartary buckwheat. The primary mechanism for obtaining water relies on the root systems of plants, necessitating a sturdy root structure for effective water absorption. A growing body of research indicates that colonization by *S. indica* can alter the shape of roots, thereby enhancing the overall surface area and root hair density (Hosseini et al., 2017; Hosseini et al., 2018; Mani et al., 2023). These results were also corroborated by our study. Furthermore, research indicates that numerous microorganisms, which enhance plant growth, are capable of triggering ABA production in leaves during drought, controlling the opening and closing of stomata, lowering transpiration rates, and reducing water loss (Cho et al., 2008; Chieb and Gachomo, 2023). Our research revealed a notable decrease in the stomatal density of tartary buckwheat leaves in the inoculated groups, in contrast to the control group (Figure 9C). Additionally, there was a notable decrease in the stomatal conductance and transpiration rates of tartary buckwheat leaves in the T15, T20, and T24 groups. This phenomenon might be attributed to the stimulation of abscisin acid (ABA) production in tartary buckwheat leaves by these microbes, which leads to stomatal closure.

Stress from drought interferes with the metabolic processes of plant cells, causing oxidative stress and an overproduction of reactive oxygen species (ROS), such as H_2_O_2_ and O_2_^−^. Such reactive oxygen species (ROS) pose a toxic threat to cells, potentially damaging proteins, lipids, and DNA. To assess oxidative stress levels, the H_2_O_2_ and MDA levels in tartary buckwheat leaves were measured during periods of drought. The findings indicated a notable reduction in H_2_O_2_ and MDA levels in the foliage of microbial consortia compared to the control and the group receiving only *S. indica* inoculation, suggesting milder drought stress, as supported by their phenotypic data. Under drought conditions, plants trigger their antioxidant defense mechanisms, enhancing the production of osmotic regulators and antioxidant enzymes (Cechin et al., 2006). The research indicated that, in contrast to the microbial consortia group, both the control group and the group inoculated solely with *S. indica* showed elevated proline and GSH levels in buckwheat leaves, along with heightened activity of enzymes such as POD, T-SOD, CAT, and GSH-Px. Likewise, research indicated that using four plant growth-enhancing bacteria together significantly boosted proline levels and antioxidant enzyme activity in barley leaves (Ferioun et al., 2024). A multitude of research indicates that *S. indica* may boost plant resistance to drought by enhancing antioxidant enzyme activity (Sun et al., 2010; Yaghoubian et al., 2014; Xu et al., 2017), a phenomenon also observed in our investigation. Relative to the control group, sole inoculation with *S. indica* markedly increased the efficacy of these antioxidant enzymes. Nonetheless, the microbial consortia group did not exhibit any increase in antioxidant enzyme activity. Our research leads us to theorize that the microbial groups in tartary buckwheat mitigated drought stress via two primary mechanisms: first, by fostering root development and boosting water absorption; and second, by enhancing photosynthesis, controlling the opening and closing of stomata, and decreasing transpiration, thus improving water efficiency. Additionally, groups of microbes could enhance tartary buckwheat’s ability to adapt to drought by controlling the production of plant hormones (such as ABA and JA), activating genes resistant to drought (Zheng et al., 2023), and engaging in ROS defense signaling pathways and the creation of osmoregulatory agents (Mamun et al., 2024). Additional studies are needed to understand their precise mechanisms.

## 5 Conclusion

**Figure 11:**
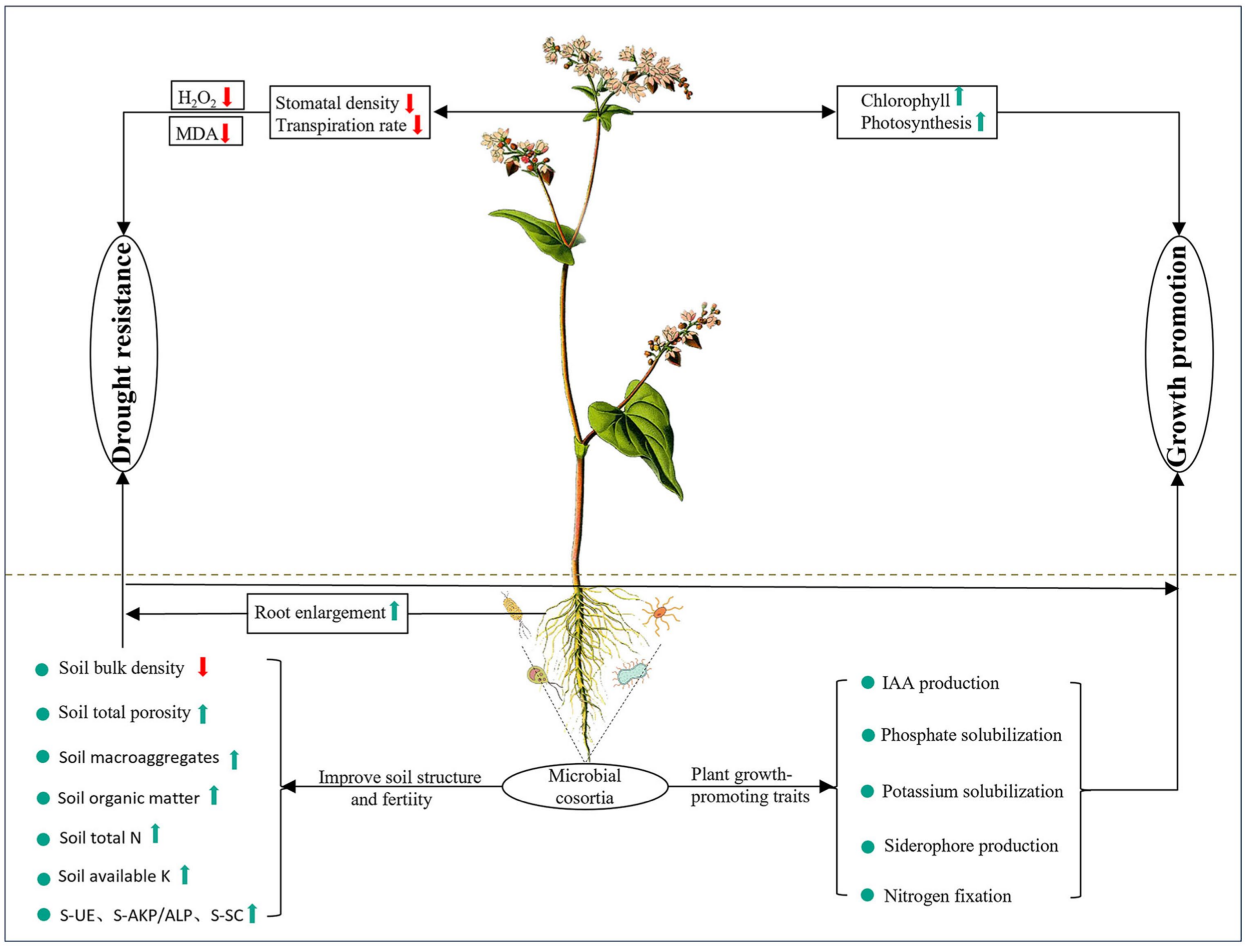
Summary diagram for the promotive effects of the microbial consortia on tartary buckwheat growth. Green and red arrows indicate parameters that increased and decreased after inoculation, respectively.

The growth and environmental adaptation of plants bear a close resemblance to microorganisms associated with roots. The impact of vaccinating against *Serendipita indica* and various microbial groups on the development of tartary buckwheat is diverse (Figure 11). This research revealed that *S. indica* has a well-established microbial composition in tartary buckwheat, enhancing the presence of beneficial microbes such as Actinobacteria, *Sphingomonas*, and *Mortierella*, while reducing the prevalence of harmful bacteria like *Cladosporium*, *Alternaria*, and *Acremonium*. Following this, we developed microbial groups by merging *S. indica* with strains of plant growth enhancers derived from wild buckwheat. Findings revealed that the presence of microbial groups markedly boosted the growth of roots and leaves in tartary buckwheat, as well as its photosynthetic abilities, in contrast to the control group. Additionally, in contrast to the control and *S. indica*-only inoculation, the presence of microbial groups markedly boosted tartary buckwheat’s resistance to drought by improving root water absorption and decreasing leaf transpiration. Furthermore, groups of microbes enhanced the composition and fertility of the soil, as well as encouraged the cycling of nutrients within it. This study’s findings offer essential theoretical backing for creating biofertilizers based on microbial consortia and aid in advancing sustainable agricultural practices.

## Data Availability

The raw data supporting the conclusions of this article will be made available by the authors, without undue reservation.

## Footnotes

^1^ http://www.arb-silva.de

^2^ https://unite.ut.ee/
