# Supplementary material for: Serendipita indica-dominated synthetic microbial consortia enhanced tartary buckwheat growth and improved its tolerance to drought stress
Source: Front Microbiol. 2025 Mar 19;16:1562341. doi: 10.3389/fmicb.2025.1562341 (PMC11961947; doi:10.3389/fmicb.2025.1562341)
Supplement: Supplementary file 1 [file Table_1.DOCX]

Supplementary Material

**Table of contents:**

**Fig S1:** Strains used in the experiments.

**Fig S2:** Non-colonized (A) and S. indica-colonized (B) detection result. The green arrow represents the chlamydospores of S. indica.

**Fig S3:** NMDS analysis of tartary buckwheat rhizosphere soil bacteria (a), fungi (b) and endophytic fungi (c).

**Table S1:** The result of strains compatibility test.

**Table S2:** The result of strain combinations.

**Table S3:** The effects of microbial consortia on the growth parameters of tartary buckwheat.


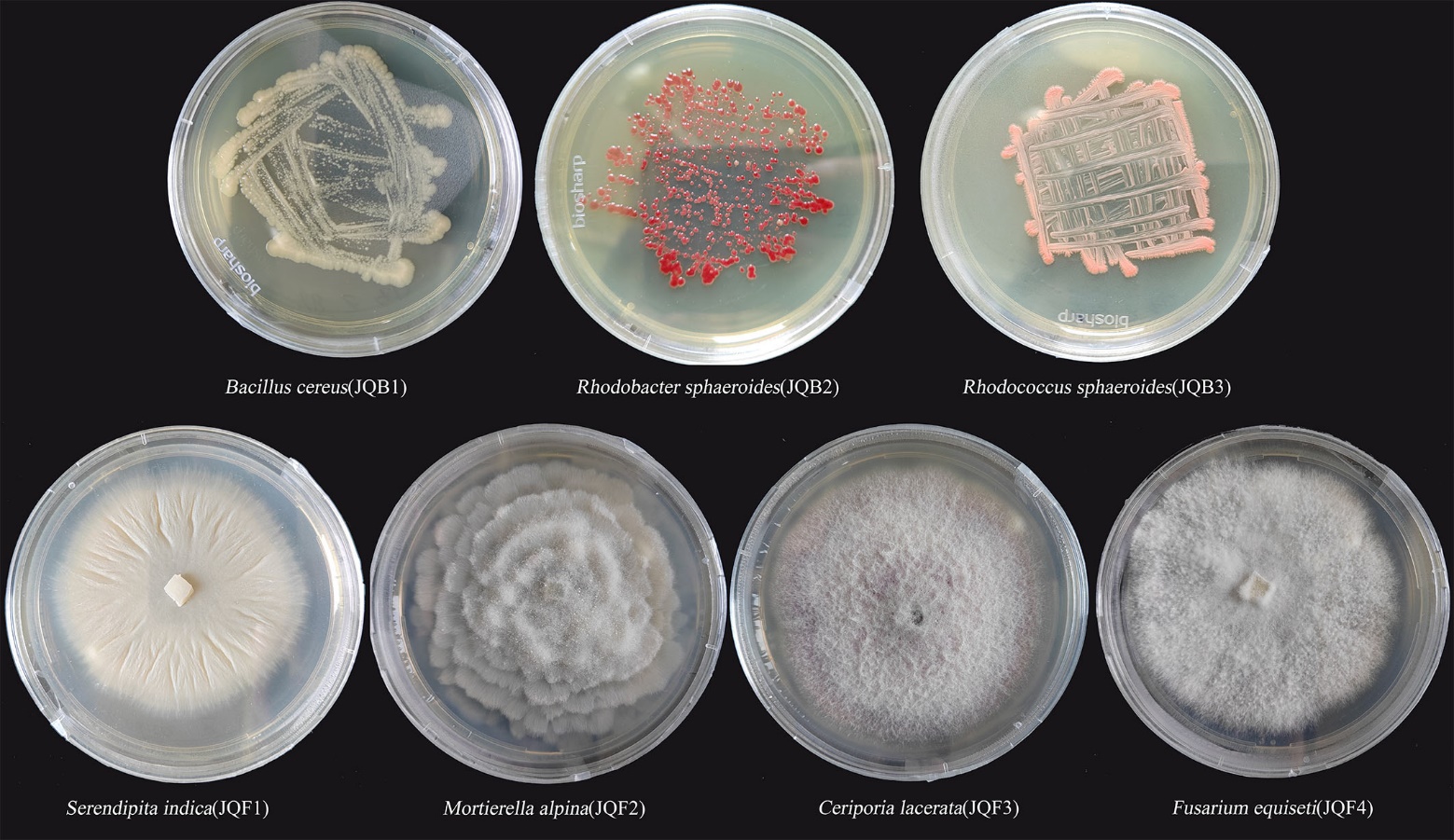


**Fig S1.** Strains used in the experiments


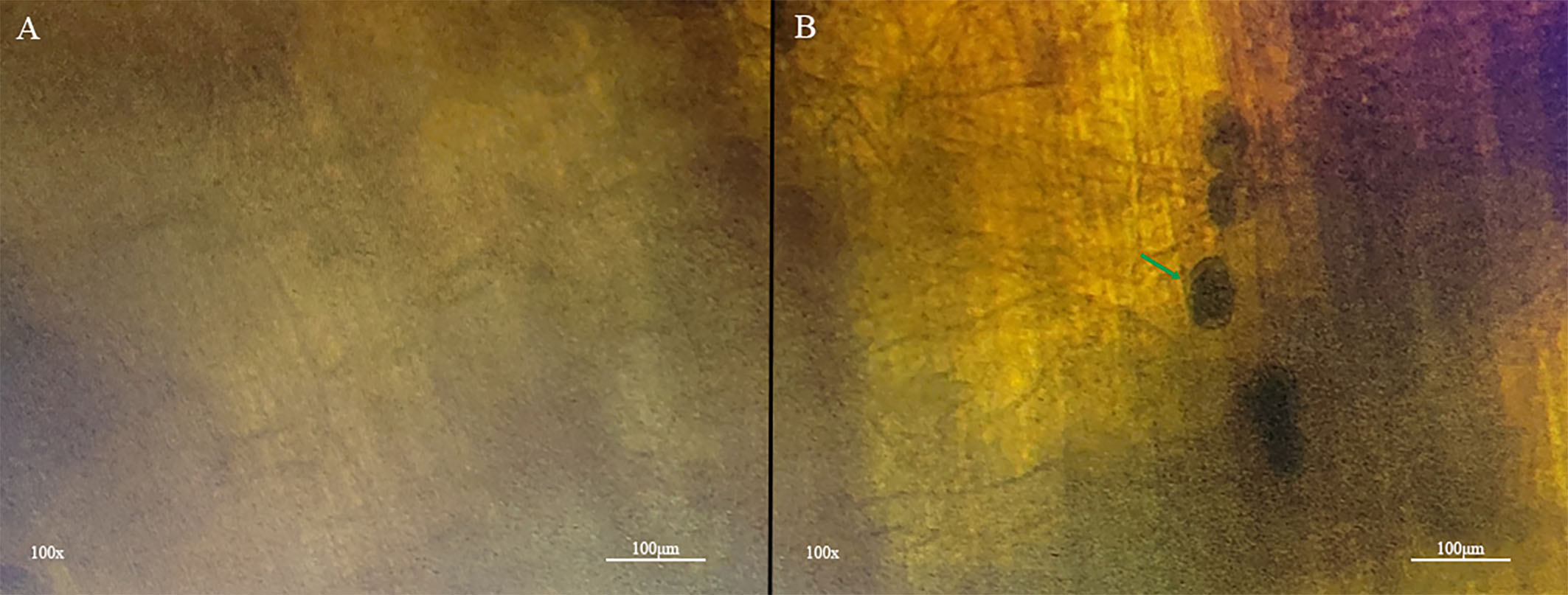


**Fig S2.** Non-colonized **(A)** and S. indica-colonized **(B)** detection result. The green arrow represents the chlamydospores of S. indica.

**
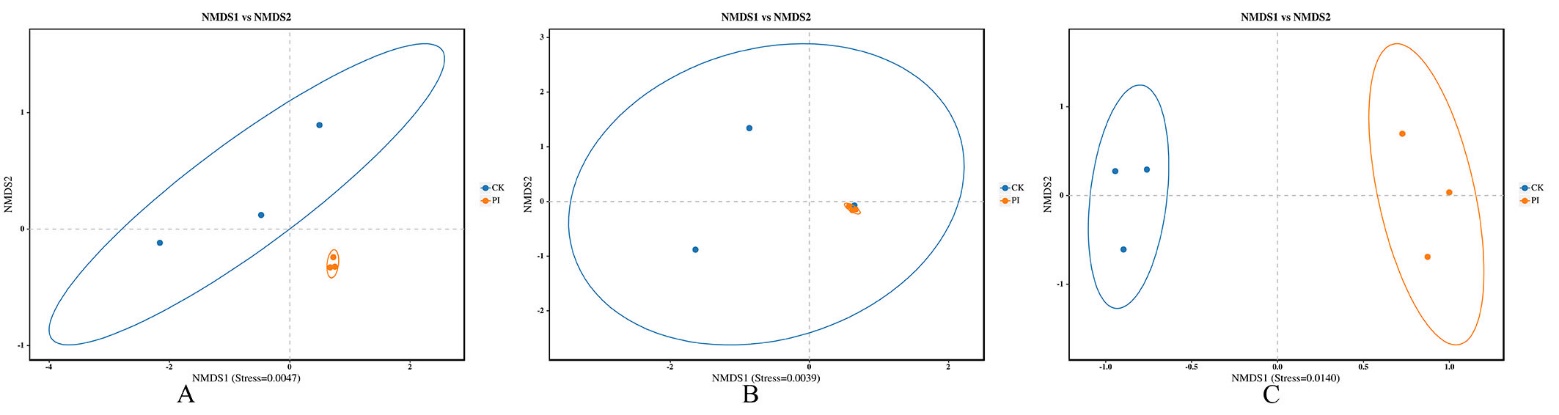
**

**Fig S3.** NMDS analysis of tartary buckwheat rhizosphere soil bacteria **(A)**, fungi **(B)** and endophytic fungi **(C).**

**Table S1.** The result of strains compatibility test.

| **Strains** | **JQB1** | **JQB2** | **JQB3** | **JQF1** | **JQF2** | **JQF3** | **JQF4** |
| --- | --- | --- | --- | --- | --- | --- | --- |
| JQB1 |  | × | √ | √ | √ | √ | √ |
| JQB2 | × |  | √ | × | × | × | × |
| JQB3 | √ | √ |  | √ | √ | × | √ |
| JQF1 | √ | × | √ |  | √ | √ | √ |
| JQF2 | √ | × | √ | √ |  | × | √ |
| JQF3 | √ | × | × | √ | × |  |  |
| JQF4 | √ | × | √ | √ | √ | × |  |

| **Number of strains** | **Treatments** | **Combination** | **Number of strains** | **Treatments** | **Combination** |
| --- | --- | --- | --- | --- | --- |
| / | CK | / | Three | T13 | JQF1+ JQB1+ JQB3 |
| Single | T1 | JQB1 |  | T14 | JQF1+ JQB1+ JQF2 |
|  | T2 | JQB2 |  | T15 | JQF1+ JQB1+ JQF3 |
|  | T3 | JQB3 |  | T16 | JQF1+ JQB1+ JQF4 |
|  | T4 | JQF1 |  | T17 | JQF1+ JQB3+ JQF2 |
|  | T5 | JQF2 |  | T18 | JQF1+ JQB3+ JQF4 |
|  | T6 | JQF3 |  | T19 | JQF1+ JQF2+ JQF4 |
|  | T7 | JQF4 | Four | T20 | JQF1+ JQB1+ JQF2+ JQF4 |
| Two | T8 | JQF1+JQB1 |  | T21 | JQF1+ JQB3+ JQF2+ JQF4 |
|  | T9 | JQF1+ JQB3 |  | T22 | JQF1+ JQB1+ JQB3+ JQF2 |
|  | T10 | JQF1+ JQF2 |  | T23 | JQF1+ JQB1+ JQB3+ JQF4 |
|  | T11 | JQF1+ JQF3 | Five | T24 | JQF1+ JQB1+ JQB3+ JQF2+ JQF4 |
|  | T12 | JQF1+ JQF4 | / | / | / |

**Table S2.**The result of strain combinations.

**Table S3.** The effects of microbial consortia on the growth parameters of tartary buckwheat. Bold values represent the group with the best growth performance among the combinations of different strain quantities. Different letters indicate significant differences (n=10, p<0.05).

| **Grop** | **Plant height(cm)** | **Leaf length(cm)** | **Leaf width(cm)** | **Petiole length(cm)** | **Stalk fresh weight(g)** | **Stalk dry weight(g)** | **Root fresh weight(g)** | **Root dry weight(g)** |
| --- | --- | --- | --- | --- | --- | --- | --- | --- |
| CK | 36.0^jk^ | 4.0^i^ | 4.5^ij^ | 3.3^j^ | 2.016^ijk^ | 0.185^j^ | 0.116^j^ | 0.019^k^ |
| T1 | 36.4^jk^ | 5.4^bcde^ | 5.3^gh^ | 4.7^defgh^ | 2.019^ijk^ | 0.187^j^ | 0.136^ghij^ | 0.022^jk^ |
| T2 | 38.0^hij^ | 5.6^abcd^ | 5.3^gh^ | 4.7^defgh^ | 2.809^efghi^ | 0.225^ij^ | 0.229^bcd^ | 0.025^hijk^ |
| T3 | 36.9^jk^ | 5.4^bcde^ | 4.9^hij^ | 4.5^efgh^ | 2.264^ghij^ | 0.190^j^ | 0.156^fghij^ | 0.022^jk^ |
| **T4** | **40.8^gh^** | **5.9^ab^** | **7.0^abcd^** | **4.7^defgh^** | **1.63^k^** | **0.192^j^** | **0.211^cde^** | **0.021^jk^** |
| T5 | 37.4^ij^ | 5.0^efg^ | 5.3^gh^ | 4.5^fghi^ | 1.396^k^ | 0.187^j^ | 0.130^ij^ | 0.022^jk^ |
| T6 | 34.3^k^ | 5.4^bcde^ | 6.5^cde^ | 4.8^cdefg^ | 1.624^jk^ | 0.182^j^ | 0.131^hij^ | 0.022^jk^ |
| T7 | 38.5^hij^ | 5.4^bcde^ | 6.9^bcd^ | 5.3^bcde^ | 1.635^jk^ | 0.213^ij^ | 0.122^j^ | 0.021^jk^ |
| T8 | 39.8^hi^ | 5.9^ab^ | 7.0^abcd^ | 4.7^defgh^ | 1.63^k^ | 0.192^j^ | 0.211^cde^ | 0.021^jk^ |
| **T9** | **47.8^cd^** | **5.7^abcd^** | **7.5^ab^** | **5.4^abcd^** | **4.483^a^** | **0.509^a^** | **0.434^a^** | **0.070^a^** |
| T10 | 54.4^a^ | 5.0^efg^ | 5.6^fgh^ | 4.6^efgh^ | 3.281^cdef^ | 0.365^bcd^ | 0.237^bc^ | 0.036^def^ |
| T11 | 48.5^c^ | 5.0^efg^ | 6.0^efg^ | 5.0^cdefg^ | 3.111^cdefg^ | 0.372^bc^ | 0.215^cde^ | 0.050^bc^ |
| T12 | 40.7^gh^ | 4.7^fg^ | 6.0^efg^ | 5.0^cdefg^ | 3.094^cdefg^ | 0.323^defg^ | 0.239^bc^ | 0.052^b^ |
| T13 | 46.5^cde^ | 5.8^abc^ | 7.6^ab^ | 6.1^a^ | 3.624^bcde^ | 0.345^bcde^ | 0.262^b^ | 0.043^cd^ |
| T14 | 45.7^cde^ | 4.9^efg^ | 6.0^efg^ | 5.0^cdefg^ | 3.175^cdef^ | 0.283^gh^ | 0.160^fghij^ | 0.033^efghi^ |
| **T15** | **45.2^def^** | **6.1^a^** | **7.7^a^** | **6.0^ab^** | **3.937^bc^** | **0.305^efg^** | **0.269^b^** | **0.043^cd^** |
| T16 | 47.5^cde^ | 5.3^cdef^ | 6.6^cde^ | 5.6^abc^ | 2.448^fghij^ | 0.310^efg^ | 0.183^defg^ | 0.033^efghi^ |
| T17 | 46.2^cde^ | 5.7^abcd^ | 7.1^abc^ | 5.8^ab^ | 3.773^bcd^ | 0.335^cdef^ | 0.233^bc^ | 0.043^cd^ |
| T18 | 44.9^ef^ | 5.1^defg^ | 6.7^cde^ | 5.6^abc^ | 3.119^cdefg^ | 0.285^gh^ | 0.160^fghij^ | 0.035^defg^ |
| T19 | 51.3^b^ | 4.6^gh^ | 5.0^hij^ | 4.4^ghi^ | 2.908^defgh^ | 0.289^fg^ | 0.179^efgh^ | 0.035^defg^ |
| **T20** | **46.6^cde^** | **5.9^ab^** | **6.3^de^** | **6.0^ab^** | **3.038^defgh^** | **0.323^defg^** | **0.234^bc^** | **0.039^de^** |
| T21 | 40.4^gh^ | 4.7^fg^ | 6.1^ef^ | 4.9^cdefg^ | 2.579^fghi^ | 0.225^ij^ | 0.152^fghij^ | 0.024^ijk^ |
| T22 | 40.5^gh^ | 4.5^ghi^ | 5.0^hij^ | 3.8^ij^ | 2.204^hijk^ | 0.241^hi^ | 0.178^efgh^ | 0.027^fghijk^ |
| T23 | 40.2^gh^ | 5.1^defg^ | 5.1^hi^ | 5.2^bcdef^ | 2.404^fghij^ | 0.222^ij^ | 0.171^efghi^ | 0.029^fghij^ |
| **T24** | **42.8^fg^** | **4.7^fg^** | **5.4^gh^** | **4.1^hi^** | **2.425^fghij^** | **0.193^j^** | **0.192^cdef^** | **0.026^ghijk^** |
